# Quality and bias of protein disorder predictors

**DOI:** 10.1038/s41598-019-41644-w

**Published:** 2019-03-26

**Authors:** Jakob T. Nielsen, Frans A. A. Mulder

**Affiliations:** 1 Interdisciplinary Nanoscience Center (iNANO), Aarhus University, Gustav Wieds Vej 14, 8000 Aarhus C, Denmark; 2 Department of Chemistry, Aarhus University, Langelandsgade 140, 8000 Aarhus C, Denmark

## Abstract

Disorder in proteins is vital for biological function, yet it is challenging to characterize. Therefore, methods for predicting protein disorder from sequence are fundamental. Currently, predictors are trained and evaluated using data from X-ray structures or from various biochemical or spectroscopic data. However, the prediction accuracy of disordered predictors is not calibrated, nor is it established whether predictors are intrinsically biased towards one of the extremes of the order-disorder axis. We therefore generated and validated a comprehensive experimental benchmarking set of site-specific and continuous disorder, using deposited NMR chemical shift data. This novel experimental data collection is fully appropriate and represents the full spectrum of disorder. We subsequently analyzed the performance of 26 widely-used disorder prediction methods and found that these vary noticeably. At the same time, a distinct bias for over-predicting order was identified for some algorithms. Our analysis has important implications for the validity and the interpretation of protein disorder, as utilized, for example, in assessing the content of disorder in proteomes.

## Introduction

Interest in intrinsically disordered proteins (IDPs) has grown immensely over the past decades. IDPs can serve a large range of functions due to their enhanced sampling of conformational space compared to structured proteins and their involvement in many important biological processes and diseases have been discovered recently^1–7^. Although experimental characterization of IDPs is very challenging, protein sequence composition has distinct biases and this has inspired the development of a large number of computational methods for predicting disorder from sequence^8,9^. Recently, predictions of disorder by various methods have been compiled into databases^10–12^ enabling consensus predictions, and meta-methods have emerged that predict disorder based on output from other predictors^13–15^. Protein disordered region (DR) prediction has been assessed periodically through the critical assessment of structure prediction (CASP) initiative^16^. DR predictions did not improve from CASP8 to CASP9^17^, and only slightly for CASP10^18^. This apparent stagnation in accuracy of disorder predictors would suggest that development of new more sophisticated predictors would not have sufficient merit, and DR predictions were not evaluated anymore in subsequent CASP assessments.

We argue that this stagnation can be attributed to the vague authority of the evaluation, caused by insufficient quality of the data used to evaluate (and train) the predictors: In CASP, DR predictors were evaluated using missing density in X-ray structures as the disorder criterion. However, regions in X-ray structures might falsely appear ordered due to biases in non-native conditions required for X-ray crystallography characterization. In addition, since only proteins amenable to X-ray diffraction are included, such data sets are imbalanced in the sense that missing residues are relatively rare (only 2.4% in the set analyzed here) causing balance problems in the model building. As a complement, disorder analysis can be done for proteins in solution, as done in the DisProt database^19,20^, and this data collection has frequently been used to train and evaluate disorder predictors^21^. Unfortunately, DisProt suffers from a heterogeneous compilation of data from diverse experimental sources, such as CD and sensitivity to proteolytic degradation, which lack position-specific information. Several false positive IDPs were indeed found in DisProt in a previous analysis^22^. A more serious issue arises from the fact that all currently applied evaluation criteria are binary classifiers, which ignore meaningful, intermediate order or a continuous range of structure^1,23,24^, and therewith limit disorder prediction to a low-precision binary-classification problem. A more balanced dataset with a higher precision and accuracy would renew the potential in the development of bioinformatics methods for predicting disorder from sequence. For this purpose, we resorted to experimental data from NMR spectroscopy.

It is well-established that proteins can be studied with high accuracy in solution under near-native conditions by NMR spectroscopy. First, the structure-determination process provides an ensemble of structures where each model is consistent with the experimental data^25–29^. Second, and more quantitatively, nuclear spin relaxation rates provide information about the time-scale and amplitude of dynamics in proteins^30–32^, capturing and validating the variability in the NMR structures. Unfortunately, spin relaxation experiments and data analysis are relatively complicated to pursue, are not applicable for all time scales or for IDPs, and therefore there is very little data available for highly dynamic sites in proteins^33^. Thirdly, chemical shifts are very sensitive to the local structure, are measured routinely and with very high precision for both structured proteins and IDPs^34,35^, and have been used extensively to report on protein structure and dynamics^36,37^. In particular, chemical shifts and their deviation from random coil values have been used to determine and quantify order/disorder and conformational propensities in IDPs^38–43^. Modern molecular dynamics (MD) simulations reproduce experimental dynamical data with increasing accuracy^44^ and, in particular, spin relaxation data has been used as an exquisite standard to benchmark MD force fields^45^. IDPs can be simulated with high accuracy in the description of local conformational equilibria, and a very close agreement has been established between the degree of order/disorder in IDPs and secondary chemical shifts^46–48^.

Recently, we introduced the Chemical shift Z-score for assessing Order/Disorder (the CheZOD score)^22^, which is based on deviations from random coil chemical shifts (RCCSs) using our refined formulation of RCCS reference values^49^. In contrast to other methods for describing order/disorder, this CheZOD Z-score provides a position-specific and continuous measure of order/disorder in proteins. Furthermore, the corresponding CheZOD database of such Z-scores for 117 proteins studied at near-native conditions is diverse and balanced, containing equal amounts of disordered and ordered residues^22^. Here, we rigorously benchmark the performance of 26 disorder prediction methods by assessing the agreement between the estimated probabilities of disorder and the experimental Z-scores for each predictor, and use this result to rank the accuracy of the predictors. We observed that the accuracy of the predictors depends on the type of features applied, the method of optimization, and that the newest predictors are generally the most accurate. Some predictors are biased towards over-predicting order. Our analysis suggests that current DR predictions are limited by the quality of the training data rather than by the capacity of the data mining approaches. Improved predictors can therefore be anticipated.

## Results

### Measures of disorder and flexibility in protein structures: p53 as an example

**Figure 1:**
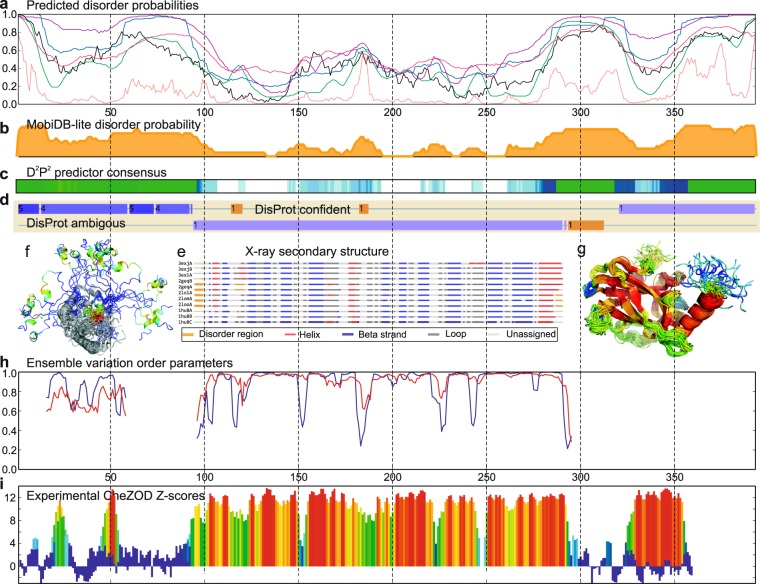
p53 experimental and inferred disorder. (**a**) Predicted disorder for PrDOS (green), IUPred_short (black), MetaDisorderMD2 (blue), RONN (pink), DISPROT_(VSL2b) (purple) and DISOPRED2 (light red). (**b**) Disorder probabilities from MobiDB-lite. (**c**) Agreement between disorder predictions from the D^2^P^2^ database shown as color intensity in a gradient bar. The green bars encode predicted disorder in segments outside predicted SCOP domains. The blue segments are where the disorder predictions intersect the SCOP domain prediction. (**d**) Inferred disorder/order from DisProt showing” disorder” and “context-dependent” regions with light brown and purple, respectively. (**e**) Assigned secondary structures for aligned chains with at least 95% sequence identity to 2FEJ analyzed and displayed using the PDBFlex server^75^. (**f**,**g**) NMR ensemble structure for the core domain (**g**) and N-terminal (**f**) as above colored according to CheZOD Z-scores as in (**i**). (**h**) NMR ensemble variation for p53 core domain (pdb id 2FEJ)^76^ and N-terminal residues 14–60 bound to HMGB1^77^ (pdb id 2lya, chain B) using red and blue lines for coordinate and angle order parameters, respectively, (see Online Methods). See also Supplementary Fig. S5 for more Z-score/flexibility protein profiles. (**i**) Experimental CheZOD Z-scores for p53 using previously assigned chemical shifts for res. 82–360^52^, data for N-term res. 1–92 from Fersht *et al*.^78^ and res. 14–60 bound to HMGB1 (in the background) as in (**g**) are shown superimposed. Z-scores are displayed with bars colored from blue through green through yellow to red indicating the highest scores corresponding to ordered residues.

To illustrate the process of disorder assignment, we consider the human oncogene protein p53, which contains ordered as well as disordered domains and is often used for illustrating predictions of disorder and interactions in IDPs^50,51^. p53 is interesting because of its involvement in more than 50% of human cancers and many diverse biological processes due to its multitude of conformations^46–48^. Estimated disorder probabilities for a large number of prediction methods (Fig. 1a, obtained from the genesilico server^13^) show agreement for some regions, but also substantial differences between the individual predictors. It is not possible to identify the most appropriate predictor *a priori* although that choice would have a dramatic impact for the prediction of disordered regions (see Supplementary Fig. S1 for prediction examples for 5 additional proteins). Consensus predictions from MobiDB-lite^11^ (Fig. 1b) and D^2^P^2 ^^10^ (Fig. 1c) suggest disorder outside of the structured domains and higher probability of disorder for the loops in the core domain (e.g. res. 181–191). However, disorder is also predicted for part of a rigid internal beta-strand in the core domain (res. 156–162) and for the entire folded tetramerization domain. When the DisProt database^20^ (Fig. 1d) is used to assign disorder, two loop regions are assigned as *confident* disorder (res. 114–120 and 182–187), whereas the linker between the core domain and tetramerization domain (res. 293–312) shows *ambiguous* disorder. The remaining residues are classified as *context-dependent*, meaning that these regions cannot be assigned unequivocally to a disordered/ordered state. X-ray structures for the p53 core domain have missing densities for the ends of some of the sequence constructs. In contrast, internal residues with missing densities were only observed for two of the 12 chains for the loop comprising residues Lys120 and Ser121, which were also classified confidently as disordered in the DisProt database (Fig. 1e). A continuous measure for local disorder/order, for which data is more abundant and balanced, is the local structural variation in an NMR ensemble. Here we introduce two types of structural order parameters, *S* and *T*, based on NMR ensemble variation in dihedral angles and the Cα internal distances, respectively, (see Online Methods). These order parameters span from zero to unity, ranging from complete disorder to order, and are in qualitative agreement with disorder predictions (e.g. for the two confident DisProt disorder regions) and show dips in order/increase in flexibility in all the loop regions of the core domain (Fig. 1h). Finally, we provide experimental disorder through the introduction of a continuous site-specific descriptor derived from assigned chemical shifts^22^ for p53^52^ (see Fig. 1i). According to these Z-scores, the core domain and tetramerization domain are ordered, whereas several loops in the core domain are disordered to varying degree (Fig. 1i). For example, the loop comprising residues Lys120 and Ser121. There is a very close agreement between disorder from Z-scores and structural flexibility in the NMR ensemble (Fig. 1f,g). A more comprehensive systematic comparison, for a large set of proteins, reveals good agreement between CheZOD Z-scores and other measures of disorder, including structural variability in MD simulations^53,54^ (see Supplementary Results 1 and Supplementary Figs S2–S5).

### Benchmarking the performance of disorder predictors

**Figure 2:**
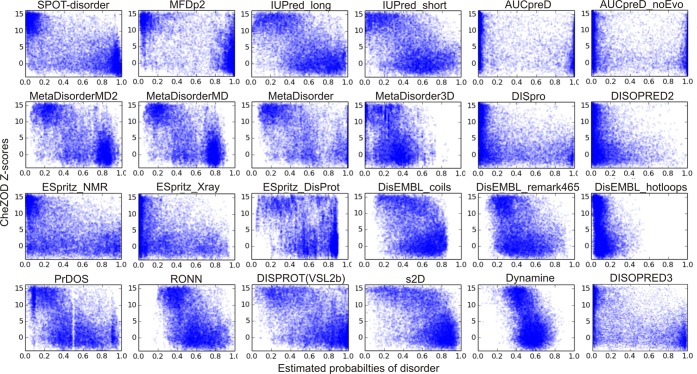
Z-score vs. estimated probability of disorder (p) for 24 continuous-valued prediction methods.

**Figure 3:**
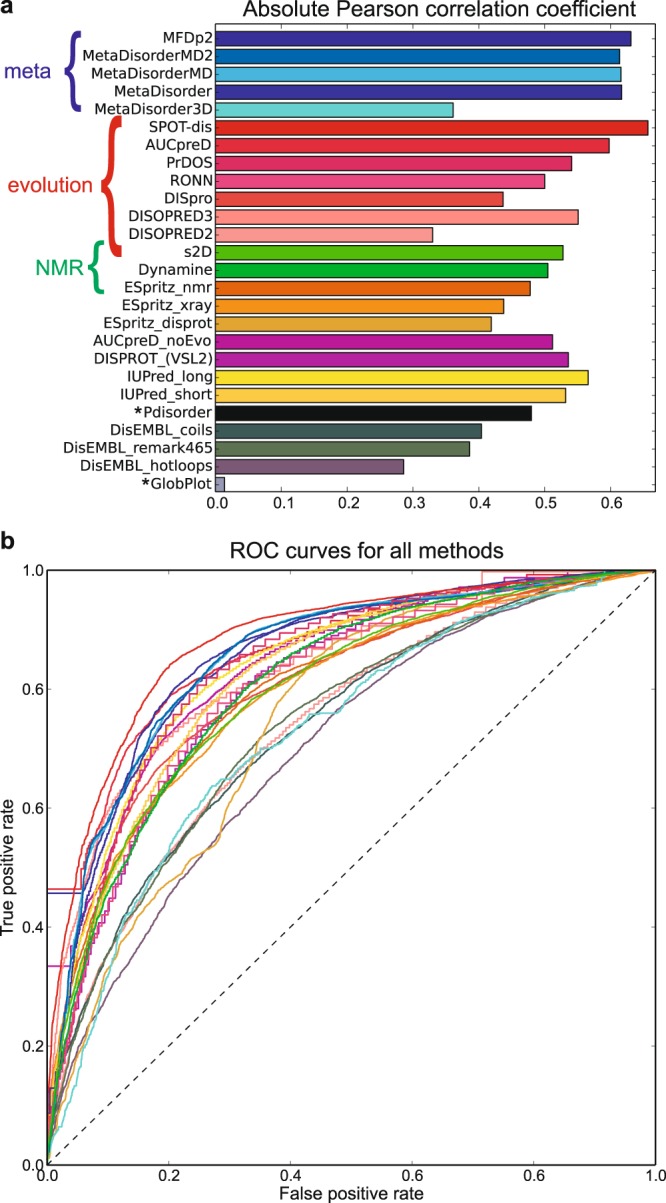
(**a**) Ranking of disorder prediction methods according to the absolute Pearson linear correlation coefficient between estimated disorder probability and Z-score shown as a histogram. The order of the methods is as in Table 1 (see Supplementary Fig. S6 for Spearman correlation). Annotation with colored curly brackets highlight meta-methods (meta), methods that apply information from evolutionary profiles of aligned sequences (evolution), and methods that use NMR data for training (NMR). Asterisks mark the binary prediction methods. (**b**) Receiver-Operating Characteristics (ROC) curves for all non-binary predictors for using estimated disorder probability to predict Z-score under/above the threshold Z = 8. Colors as in the histogram. The corresponding area under the curve (AUCs) are provided in Table 1 and shown as histograms in Supplementary Fig. S7. Note that the edgy appearance of some of the ROC curves are due to fewer decimal points on the estimated probabilities of disorder.

Above, a qualitative agreement was observed between Z-scores and estimated disorder probabilities for p53 with some noteworthy differences between individual predictors. To analyze the agreement systematically, disorder predictions were obtained for the 117 proteins in the CheZOD database as described in Online Methods. The calculated Z-scores were compared to the estimated disorder probabilities for a large set of different disorder predictors (see Table 1 and Online Methods) with the aim of identifying the best methods as those having the best agreement between estimated disorder probabilities and Z-scores. Figure 2 shows scatter plots of the Z-scores vs. the estimated probabilities (Z vs. p) for each predictor. It is seen that most predictors provide relatively high estimated probabilities of disorder for residues with low Z-scores and correspondingly lower probabilities for residues with high Z-scores. Qualitative agreement is observed, but the predictions are clearly different, with different qualities and biases in the correlation with Z-scores. To assess this agreement quantitively, we take full advantage of the continuous descriptor of disorder by determining the Pearson correlation coefficient, R_P_, of agreement (see Fig. 3). This number is ideal for ranking the predictors from the best (largest absolute value) to the worst. As Z-scores increase with *order* while *p* is a measure of *disorder*, –1 indicates a perfect correlation and 0 expresses a complete lack of correlation. It is seen that binary predictors show poor correlation, while the newer, continuous methods SPOT-disorder^55^, MFDp2^14^ and AUCpreD^56^ predict best (Table 1 and Fig. 3). Furthermore, the genesilico metapredictors^13^ perform slightly better than all the methods used by the metapredictors but slightly inferior to the newer methods mentioned above (Table 1 and Fig. 3). The ESpritz^57^ methods perform increasingly well, when trained on DisProt data, X-ray data, and NMR data, respectively (Table 1 and Fig. 3). Two methods that use NMR data for training – s2D^58^ and DynaMine^59^ – were also included. These methods were trained on continuous-valued target data; i.e. chemical shift derived secondary structure populations for *s2D* and local fast dynamics, as defined by the order parameter, for DynaMine. Here we interpret the predicted populations of non-alpha-helix/beta-sheet as the probability of disorder and use a bijective transformation of the predicted order parameters to convert it to a pseudo-probability (see Online Methods). Judged by the Pearson correlation coefficient, these two methods are ranked in the middle for predicting Z-scores. The Spearman rank correlation coefficient, *R*_*S*_, describing the agreement with a monotonic relationship between *p* and *Z* (not necessarily linear) was also calculated, and showed the same trend for the predictors (see Table 1 and Supplementary Fig. S6).

**Table 1:** Performance of disorder predictors.

Method	R_P_	R_S_	AUC	pZA	pZD	Input^a^	Class^b^
MFDp2	−0.631	−0.592	0.853	0.582	0.490	Pdis	Meta
MetaDisorderMD2	−0.614	−0.579	0.852	0.513	0.325	Pdis	Meta
MetaDisorderMD	−0.616	−0.580	0.853	0.479	0.308	Pdis	Meta
MetaDisorder	−0.617	−0.575	0.865	0.590	0.399	Pdis	Meta
MetaDisorder3D	−0.361	−0.352	0.727	0.261	0.126	ST	ML
SPOT-dis	−0.657	−0.638	0.881	0.426	0.475	Evo	ML
AUCpreD	−0.598	−0.588	0.865	0.441	0.552	Evo	ML
PrDOS	−0.541	−0.543	0.836	0.403	0.277	Evo/ST	ML
RONN	−0.500	−0.495	0.804	0.525	0.172	Evo	ML
DISpro	−0.437	−0.498	0.805	0.221	0.269	Evo	ML
DISOPRED2	−0.330	−0.404	0.738	0.120	0.109	Evo	ML
DISOPRED3	−0.551	−0.553	0.833	0.332	0.388	Evo	ML
s2D^c^	−0.528	−0.501	0.797	0.610	0.241	Evo	ML
Dynamine^c^	−0.505	−0.489	0.806	0.502	0.124	AA	ML
ESpritz_NMR	−0.478	−0.483	0.797	0.335	0.300	AA	ML
ESpritz_Xray	−0.438	−0.474	0.791	0.208	0.230	AA	ML
ESpritz_DisProt	−0.419	−0.374	0.748	0.575	0.209	AA	ML
AUCpreD_noEvo	−0.512	−0.552	0.841	0.386	0.460	AA	ML
DISPROT (VSL2b)	−0.536	−0.497	0.808	0.609	0.286	AA	ML
IUPred_long	−0.566	−0.541	0.834	0.493	0.302	AA	SF
IUPred_short	−0.532	−0.505	0.822	0.424	0.275	AA	SF
Pdisorder^d^	−0.480	n.a.	n.a.	0.523	0.430	AA	ML
DisEMBL_coils	−0.404	−0.364	0.735	0.523	0.150	AA	ML
DisEMBL_remark465	−0.386	−0.389	0.737	0.405	0.140	AA	ML
DisEMBL_hotloops	−0.286	−0.334	0.702	0.109	0.049	AA	ML
GlobPlot^d^	−0.014	n.a.	n.a.	0.064	0.008	AA	SF

^a^Input: AA (AA type/property and composition); Evo (evolutionary information based on multiple sequence alignment profiles); ST (Structural templates); Pdis (estimated disorder probabilities from other predictors).

^b^Class: Meta (meta-predictor); ML (machine learning); SF (scoring function).

^c^Predicts continuous-valued NMR parameters (see Methods). Since the prediction output is not an actual disorder probability, the derivation of pZA and pZH do not strictly apply (see text and Online Methods).

^d^Binary prediction methods. Derivation of Spearman correlation and AUC do not apply.

It is evident from Fig. 2 that predictions and Z-scores cluster in four quadrants due to the underlying bimodal distribution of Z-scores^22^ and the binary nature of the classification used for training the methods. A very slight over-representation of “medium-range” Z-scores (close to 8.0) for average probabilities (close to 0.5) is seen only for the best ranked methods and IUPred^60^. To enable comparison with previous benchmarks, we also performed analysis for a binary classification of disorder using the definition Z < 8 for disorder. This Z-score threshold provides the optimal agreement for a binary classification of order/disorder for all prediction methods on average (see Supplementary Fig. S10). A good predictor should optimize the fraction of correctly identified disordered residues (true positives, TP) while simultaneously minimizing the fraction of false positives (FP). ROC curves display TP vs. FP as a function of the probability threshold and the corresponding area under this curve (*AUC*) is an aggregate measure of the quality of a predictor that is not affected by any skew/bias of the estimated probabilities. A perfect classifier would yield *AUC* = 1, whereas random guessing gives *AUC* = 0.5. ROC curves for all predictors are shown in Fig. 3 and the *AUC* values are listed in Table 1. The non-binary methods display *AUCs* ranging from 0.733 (MetaDisorder3D) to 0.890 (SPOT-disorder) and reiterate the trends described above for the ranking of the predictors (see Table S1 and Supplementary Fig. S7).

**Figure 4:**
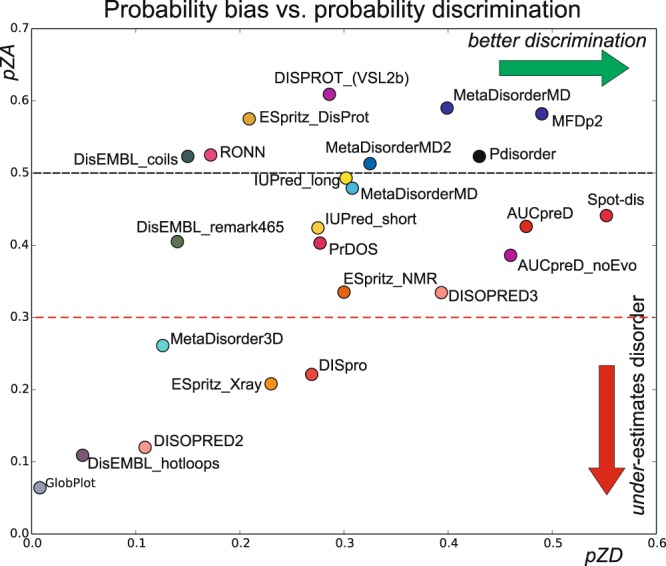
Probability bias vs. probability discrimination showing pZA as a function of pZD (see text and Online Methods). Each predictor is shown with a circle using the same colors as in Fig. 3 above. A black broken line corresponding to a completely unbiased predictor with pZA = 0.5 is shown for reference. Predictors below the red dashed line (pZA = 0.3) considerably under-estimate disorder. Methods that noticeably over-predict disorder (i.e. pZA > 0.7) were not observed.

It is apparent from Fig. 2 that some predictors are continuous, while other are more bimodal. In addition, for some methods predictions cluster on one side, suggesting a prediction bias. To quantify this bias of over-predicting order or disorder, the average probability of predicting low Z-scores (pZL for Z-scores < 8.0) and high Z-scores (pZH, Z-scores > 8.0) was calculated for each method. An unbiased method would have an average probability pZA = (pZL + pZH)/2 close to 0.5. At the same time, methods with good discrimination between order and disorder will display a large probability difference, pZD = pZL − pZH. Figure 4 plots the average probability (pZA, bias) against the probability difference. It is seen that DISOPRED2^4^, DisEMBL hotloops^61^ and GlobPlot^62^ are biased towards under-predicting disorder (e.g. using pZA < 0.3). On the other hand, no methods over-predict disorder (no method has pZA > 0.7). Along the other axis, SPOT-disorder has the highest probability difference suggesting the best (formal) discrimination between order and disorder. The above findings are mirrored in a classical confusion-based analysis (see Supplementary Table S2) except that for DISOPRED2 and DisEMBL hotloops a probability cut-off different from p = 0.5 was used, and therefore no significant over-prediction of order was found by this analysis. GlobPlot and ESpritz-Xray^57^ methods have False Negative Rates (FNRs) as high as 0.98 and 0.718, respectively, but at the other end of the extreme, the methods with the highest False Positive Rates (FPRs), ESpritz_DisProt and DISPROT^63^ (VSL2b), have FPRs of 0.415 and 0.401, respectively, and do not over-predict disorder to a similar extent.

## Discussion

Residues with missing X-ray densities are relatively rare, with only 2.4% of the residues being non-observed in the dataset tested here (see Methods) and 8.6% in a set used for training SPOT-disorder^55^ (See Supplementary Discussion and Supplementary Table S2) and the disordered regions identified in X-ray data are relatively short (Supp.Table S2). Conversely, long regions of disordered residues as well as completely disordered proteins are abundant in the DisProt database^19,20^ (see Supplementary Discussion). This pronounced difference between the two data sources has long been realized, and complementary methods dedicated to predicting either short or long regions of disorder have been developed by training on X-ray or DisProt data, respectively^57,64,65^. Interestingly, yet maybe not surprising, dedicated subversions of predictors show the best performance when evaluated on the same type of data as were used for training^21,66^. To elaborate on this, the CheZOD database was divided into different subsets chosen as to represent data sets with different characteristics as e.g. content of disorder and size of disordered regions (see Supplementary Discussion). It was found that the ranking of the prediction methods was generally preserved and that the performance on the different subsets reflect the data used for training the methods (see Supplementary Discussion and Supplementary Figs S8 and S9). Since the CheZOD database is diverse and balanced, containing both structured proteins with short and long disordered loops as well as completely disordered proteins^22^, it is ideal for assessing the performance of predictors of general disorder of no particular flavor.

NMR-derived Z-scores for proteins in the CheZOD database have been applied here in an attempt to rigorously benchmark the performance of a large number of disorder predictors (see Table 1). Contrary to CASP chronological extrapolations outlined above, it was found that the most recent predictors feature improved performance. Notably, the newer implementation of DISOPRED, DISOPRED3^67^, performs significantly better than the older version, DISOPRED2^4^. Several trends in the performance of the predictors related to the type of inputs and optimization procedure were observed. Older methods and methods that focus on speed use only amino acid (AA) sequence-based features, such as AA composition, physiochemical properties, interaction energies and sequence complexity, and display comparatively less good performance. Inclusion of evolutionary information derived from multiple sequence alignment profiles expands the repertoire with complementary features. The group of predictors here that use evolutionary (Evo) information generally perform better than the predictors without it (Table 1). Finally, the metapredictors that use estimated disorder probabilities from other predictors display very good performance.

**Figure 5:**
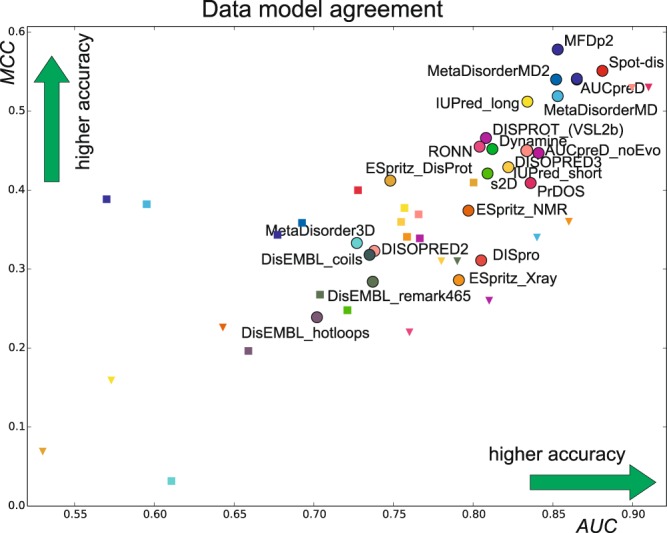
Performance of predictors on different data sets. MCC vs. AUC is shown for each method tested here with a circle using same colors as in Fig. 3 and compared to values reported in the literature for methods also tested here depicted as squares when tested against DisProt data and triangles when tested against X-ray data. See Supplementary Table S1 for all numbers and details related to DisProt and X-ray source data (Note that some of the prediction methods analyzed here were not included in the corresponding studies tested against DisProt or X-ray data, and hence, there are fewer labels of triangles and squares).

To compare the authority of different data-sources to judge disorder, we perform a comparison across data for the same methods by deriving traditional binary classifier metrics; the AUC and the Mathews correlation coefficient (MCC) (see e.g.^18^). MCC is a balanced measure of correlation that considers false and true positives as well as their negatives. The AUC and MCCs were calculated and compared to values reported in the literature for testing against DisProt^21^ and X-ray data, as summarized previously^9^ (see Table S1 and Fig. 5). We find that values for both AUC and MCC are significantly higher for the same predictors when compared to the DisProt and X-ray evaluation sets, respectively (Fig. 5 and Table S1). This strongly suggests that the CheZOD Z-score classifier is more predictable and more accurate, in the sense that it contains fewer miss-classifications.

The analysis presented here provides a guideline for selecting the most appropriate predictor for assessing disorder and to avoid intrinsic bias. As a point in case, DISOPRED2 was used to estimate the content of disordered residues in various proteomes revealing a content of ca. 33% in Eukaryotes^4^. Importantly, our analysis now shows that DISOPRED2 markedly *under*-predicts disorder, suggesting that protein disorder in eukaryotes is even more prevalent than previously assumed.

## Conclusions

We have demonstrated that validated, balanced NMR chemical shift data of proteins can be used to benchmark widely-used disorder predictors. Cross-data comparison of the performance for the same predictors demonstrated that the CheZOD dataset is more appropriate than previously utilized sources. A detailed analysis revealed that the most recent and most advanced prediction methods display the best performance, and bias for under-predicting disorder was evaluated quantitatively. We provided several performance measures to help researchers make an informed decision for selection of the most appropriate disorder prediction method.

## Methods

### Production of disorder probabilities for the proteins in the benchmarking set

The genesilico metaserver (http://iimcb.genesilico.pl/metadisorder/) was used to obtain estimated probabilities of disorder for a range of different disorder prediction methods (see Table 1 in main text) including their own meta-predictors. Furthermore, we added predictions from several other methods where parallel batch job submission was possible using their servers: SPOT-disorder^55^, MFDp2^14^, AUCpreD^56^, three versions of ESpritz^57^ based on different training data, *viz*. X-ray missing density, NMR ensemble structural disorder classification and DisProt disorder. Classic DisEMBL binary predictions were replaced by continuous predictions using the automatic job submission system at http://dis.embl.de. DynaMine^59^ and s2D^58^, which predict continuous NMR data, were also included. Predictions of populations of secondary structure types from *s2D* were interpreted using the sum of the estimated populations of alpha-helix and beta-sheet as a probability of order, as before^24^. Predictions of the order parameter *S*^2^ from *Dynamine* were converted to a probability of disorder using the bijective transformation $p=\sqrt{1-{S}^{2}}$. To summarize, the prediction methods tested were: MetaDisorder including MD/MD2/3D variants^13^, SPOT-disorder^55^, AUCpreD (with/without evolution)^56^, MFDp2^14^, PrDOS^68^, RONN^69^, DISpro^70^, DISOPRED2^4^, DISOPRED3^67^, *s2D*^58^, DynaMine^59^, ESpritz NMR/Xray/DisProt variants^57^, DISPROT^63^ (VSL2b) (also referred to as PONDR), IUPred long/short variants^60^, Pdisorder (http://www.softberry.com/), DisEMBL coils/remark465/hotloops variants^61^ and GlobPlot^62^.

### The set of structured proteins with chemical shifts

The database of structured proteins described before^49^ was used. However, in the present study we did not exclude entries homologous to proteins from the CheZOD database leading to a final set of 896 proteins with assigned chemical shifts. From this set, 222 proteins structures were determined by X-ray crystallography whereas the remaining 674 were determined by NMR spectroscopy. A trimmed unbiased set of X-ray structures was derived from the set of 222 proteins by removing entries if (i) the biologically significant oligomerization state was not a monomer, (ii) larger ligands were present, (iii) the protein sequence of the X-ray structure and the corresponding sequence of assigned chemical shifts differed for more than 10% of the residues. These criteria resulted in a reduced database of 90 entries. For both sets of X-ray structures, residues in the X-ray sequence (SEQRES record) that were absent in the coordinate section (i.e. those mentioned in the REMARK 465) were identified. Following this procedure, we identified 717 missing residues in the set of 222 X-ray structures compared to 30495 residues that were observed in the structure - and similarly 234/13581 for the reduced 90 entries set. Note that only residues with assigned chemical shifts in the corresponding NMR study were included in the above analysis. Within the set of entries corresponding to NMR structures, the 100 with the highest fraction of residues with CheZOD Z-scores < 5.0 were selected and used for comparison with the parameters (see below) describing structural variation in the corresponding NMR ensemble of structures. Furthermore, we identified 23 proteins from the refDB database^71^ described above having chemical shifts assigned for all backbone atom types that had available simulated molecular dynamics trajectories in the Dynameomics database^53,54^. The Z-scores were compared to the rms Cα coordinate fluctuations within the MD trajectories for these proteins.

### Definition of torsion angle and coordinate variations and order parameters

The dihedral angle order parameter *S*_*HW*_ of Hyberts, Wagner and co-workers^72^ is defined as:

1 $${S}_{HW}(\theta )=\frac{1}{N}\sqrt{{(\sum _{i=1}^{N}\sin ({\theta }_{i}))}^{2}+{(\sum _{i=1}^{N}\cos ({\theta }_{i}))}^{2}}$$

for an ensemble of *N* structures, where *θ*_*i*_ is the value of a particular dihedral angle *θ* in the *i*^th^ member of the ensemble. Based on the backbone dihedral angles ϕ and ψ, the sequence-specific backbone dihedral angle parameter, *D*_*i*_, for residue *i* in a protein sequence is defined as:

2 $${D}_{i}=\frac{1}{6}\sum _{j=i-1,\,i,i+1}({S}_{HW}({\varphi }_{j})+{S}_{HW}({\psi }_{j}))$$

This order parameter is converted to a torsion angle standard deviation, *s*(i), using the approximate relation^72^:

3 $$s(i)=2\arccos (1+\frac{\mathrm{ln}({D}_{i})}{2})$$

A parameter describing the variation in Cartesian coordinates for a specific residue is derived from the inter-atomic variance matrix (IVM) following a procedure akin to the FindCore algorithm^73^. Each element, *v*_ij_ in the variance matrix is defined as:

4 $${v}_{ij}=\frac{1}{N}\sum _{k=1}^{N}{({d}_{ijk}-{\bar{d}}_{ij})}^{2},\,{\bar{d}}_{ij}=\frac{1}{N}\sum _{k=1}^{N}{d}_{ijk}$$

where *d*_*ijk*_ is the Cα(i)-Cα(j) distance for conformer, *k*, in the ensemble.

Each row, v_i_, in the matrix, excluding diagonal and next-to-diagonal elements, v_ii_ and v_ij_ with |i-j| = 1 is sorted numerically and indexed by increasing rank:

5 $${\lambda }_{i1} < {\lambda }_{i2} < \cdots  < {\lambda }_{in}$$

where ${\lambda }_{ij}={v}_{iq}$ is *j*’th smallest element of the row v_i_ and *n* denotes the total number of such variance elements – i.e. the number of residues minus 3.

The residue coordinate variation, *t*(i), is then calculated as the weighted average:

6 $$t(i)=\frac{{\sum }_{j=1}^{n}{w}_{j}\sqrt{{\lambda }_{ij}}}{{\sum }_{j=1}^{n}{w}_{j}},\,{w}_{j}={e}^{-\beta {(\frac{j}{n})}^{2}}$$

where β = 10.0 is used here. The parameters, *s* and *t*, describing the residue angle and coordinate variation, respectively, are then converted to the corresponding order parameters, *S* and *T*, using:

7 $${\rm{S}}=\frac{1}{(1+{(\frac{{\rm{s}}}{{{\rm{s}}}_{0}})}^{2})}\,{\rm{a}}{\rm{n}}{\rm{d}}\,{\rm{T}}=\frac{1}{(1+{(\frac{{\rm{t}}}{{{\rm{t}}}_{0}})}^{2})}$$

where *s*_0_ = 75° and *t*_0_ = 1.5 Å were used here as the reference values.

The Jensen-Shannon divergence, JSD^74^, describes the similarity between two (discrete) probability distributions, *P* and *Q*.

8 $$JSD(P,Q)=\frac{1}{2}D(P||M)+\frac{1}{2}D(Q||M)$$

where *M* is the average of the distributions

9 $$M=\frac{1}{2}(P+Q)$$

and D is the Kullbeck-Leibner divergence:

10 $$D(P||M)=\sum _{i}P(i)\mathrm{log}(\frac{P(i)}{M(i)})$$

here we calculate JSD for the distributions of Z-scores corresponding to above/below reference values *s*_0_ = 1.5 Å and *t*_0_ = 75° for the residue angle and coordinate variation, respectively, and for residues corresponding to observed residues in X-ray structures vs. missing residues (REMARK 465).

## Supplementary information

Supplementary Information

## Data Availability

The full database containing protein sequences, BMRB id, and CheZOD Z-scores is available at http://www.protein-nmr.org./.
