# Supplementary material for: Quality and bias of protein disorder predictors
Source: Sci Rep. 2019 Mar 26;9:5137. doi: 10.1038/s41598-019-41644-w (PMC6435736; doi:10.1038/s41598-019-41644-w)
Supplement: Supplementary file 1 — Supplementary Information [file 41598_2019_41644_MOESM1_ESM.docx]

Quality and bias of protein disorder predictors

Jakob T Nielsen^1,2^* and Frans A. A. Mulder^1,2^*

^1^Interdisciplinary Nanoscience Center (iNANO) and ^2^Department of Chemistry, Aarhus University, Gustav Wieds Vej 14, 8000 Aarhus C, Denmark

*Correspondence to: jtn@inano.au.dk, [fmulder@chem.au.dk](mailto:fmulder@chem.au.dk)

# Supplementary Information

### **Supplementary Results 1**: Comparison of CheZOD Z-scores with other measures of protein structure flexibility and dynamics for a larger set of proteins

The CheZOD Z-scores from a database^1^ of proteins with known structure were compared to the frequently used criteria of disorder in X-ray structures as missing density (remark 465 in the pdb file) (see Online Methods) using Receiver Operating Curve (ROC) analysis. The ROC curves plot the fraction of correctly identified missing density cases (true positive rates) against the false positive rates, parametrized by the cutoff in Z-scores. The ROC curves are shown in Supplementary Figure S2 for all 222 proteins with X-ray structures in the database and for the 'unbiased' subset as well (90 proteins, see legend to Figure 1 and Online Methods). A perfect relationship would give an area under the ROC curve (AUC) very close to unity, whereas a random classifier would yield AUC = 0.5. As judged by an *AUC* of 0.925, the missing densities agree well with our NMR-derived Z-score criterion. More specifically, a cut-off of *Z* = 8.0 detects 79.4% of the missing residues in X-ray structure at the expense of only 4.8% of the observed residues classified falsely as non-observed (false positives). The ROC curve rises quickly to detect a moderate level, 50%, of the true positives (at a false positive rate of only ca. 1%), but does not get very close to 1.0 until a false positive rate of ca. 80%. This means that even at a relatively high threshold Z-score, there will still be a few false negatives. Such undetected missing densities could, for example, occur due to hinge movements when a protein consists of structured domains, connected by a flexible linker. Missing densities in the X-ray structure might be a result of static, rather than dynamic disorder, whereas high Z-scores for the folded domains would be obtained, because NMR chemical shifts are governed by local structure. Conversely, an intrinsically disordered part of a protein, being flexible when studied in solution, and yielding low Z-scores, might be stabilized by addition of co-factors or by packing forces in crystals, leading to false positives.

In a further validation approach, we have introduced two metrics for quantifying order/disorder through the variation in NMR ensembles (see Online Methods, Eqs. (3) and (6)): one reporting on dihedral angle variations, and another dependent on inter-model Cα-Cα distance variations. These metrics are represented by two order parameters, S and T, respectively, (Eq. (7)). In a set of 100 protein structures (see **Supplementary Figure S3**) the calculated Z-scores and the derived order metrics *S* and *T* showed Pearson correlation coefficients R of -0.490 and -0.589, respectively, and a correlation R = -0.613 for the average of the two, indicating that the variation in NMR ensembles is closely mirrored by the disorder/order inferred from chemical shifts. The distribution for the Z-scores are rather different for angle and coordinate variations when comparing them below and above threshold values *s_0_* = 75°, and *t_0_* = 1.5Å, corresponding to order parameters *S* = *T* = 0.5 (see Supplementary Figure S4). This was further quantified by use of the Jensen-Shannon divergence, *JSD* (a measure of the difference between two distributions; see Online Methods), which equaled 0.293 and 0.367 for angle and coordinate variations, respectively. The *JSD* for coordinate variation was comparable when using missing residues in X-ray structures as the split criterion (*JSD* = 0.383; note that these were derived for different datasets, however). It should, however, be noted that the merit of these order parameters is strongly dependent on the quality of the NMR ensemble, and a paucity of local constraints can inflate the apparent structural rigidity and artificially reduce order parameters On the other side, over-weighted distance constraints (due to overly powerful minimization algorithms for structure calculation) can result in a smaller variation in the ensemble structure and thereby a higher order parameter, despite disorder actually being present.

In a third analysis, the NMR Z-scores were compared to the rms coordinate fluctuations (rmsfs) for proteins within the Dynameomics set^2,3^ (see Online Methods). The rmsfs were derived for the Cα atoms, averaged over trajectory windows of 500ps. A Pearson correlation R = -0.595 was obtained in this case (see Supplementary Figure S5). Unfortunately, this set contained only very few disordered residues, since most of these were absent from the Dynameomics collection in the process of the MD workflow.

### **Supplementary Discussion**: Analysis of regions of order and disorder using subsets of the CheZOD database.

Although we argue that the assessment power of our dataset lies in the fact that the CheZOD Z-scores provide a real-valued scale for disorder, it can be insightful to coarse grain the Z-score to a binary measure of either order or disorder judging by the value of the Z-score over or below the threshold, Z = 8.0. A region is defined here as a consecutive stretch of residues classified as either all ordered or all disordered (all Z < 8.0 or Z > 8.0). This allows for an additional analysis of both the lengths and the number of regions as well as the sequence position of the borders of these regions.

### Notation of regions of order and disorder

The disordered/ordered regions in a protein are fully described by the sequence positions for the borders between regions, which we denote here as:

|  | $d_{1},d_{2},\ldots,d_{n} and o_{1},o_{2},\ldots,o_{m} with \left\vert m-n \right\vert=0 or 1$ | (S1) |
| --- | --- | --- |

where d_k_ and o_k_ denotes a border transiting from order to disorder and vice versa, respectively (note that a completely disordered protein has no borders and m = n = 0). The total number of borders is B = m + n. The average region length, *R*, in a set of proteins is defined here as the fraction between the total number of residues, *N*, in the set of proteins and total number of borders:

|  | $R=\sum_{s} N(s)/\sum_{s} B(s)$ | (S2) |
| --- | --- | --- |

where s denotes a protein sequence in the set. Similarly, a *predicted* disordered state is defined by the estimated probability: p > p_lim_. p_lim_ = 0.5 is used by default except for methods specifically mentioned in the legend to Supplementary Table S1. This leads to predicted regions of disorder and order and the predicted average region length, R’. The predicted border positions are denoted using primes (similarly to Eq. (S1)):

|  | ${d'}_{1},{d'}_{2},\ldots,{d'}_{n'} and {o'}_{1},{o'}_{2},\ldots,{o'}_{m'} with \left\vert m'-n' \right\vert=0 or 1$ | (S3) |
| --- | --- | --- |

The average border position prediction error, Δ*B*, is the difference between observed and predicted border positions calculated as an average across all borders and protein sequences, s:

|  | $\Delta B=\frac{\sum_{s} \left( \sum_{k=1}^{n(s)} \delta_{d}\left( s,k \right)+\sum_{k=1}^{m(s)} \delta_{o}\left( s,k \right) \right)}{\sum_{s} B(s)}$ | (S4) |
| --- | --- | --- |

where

|  | $\delta_{b}\left( s,k \right)=\min\left( 50,\frac{n\left( s \right)}{2},\min_{j} \left\vert b_{k}\left( s \right)-b_{j}^{'}\left( s \right) \right\vert\right)$ | (S5) |
| --- | --- | --- |

calculates the difference for the k^th^ border, b_k_(s), for either order (o) or disorder (d) for protein sequence, s. Note that for a protein sequence with exclusively single state predictions (e.g. all residues predicted to be disordered) the minimum reduces to $\min\left( 50,\frac{n\left( s \right)}{2} \right)$. Conversely, a prediction with many fluctuations below/above p_lim_ leads to many predicted borders and a larger set to the derive the right-hand side minimum from and, hence, a lower number for the border difference is to be expected.

### Disorder data source characteristics

The data sources used for judging disorder have different characteristics. CASP evaluations were judged by the missing coordinates in X-ray structures and later also from variation in NMR ensemble structures estimated using MOBI classification from NMR-derived ensemble structures^4^. Conversely, the DisProt database^5^, frequently used for training disorder predictors, relies on data from various biophysical characterization methods (including NMR spectroscopy and X-ray diffraction). The characteristics are summarized in Supplementary Table S3. Much shorter regions of disorder and average region lengths (Eq. S2) as well as smaller fractions of disordered residues are found in data sets derived from X-ray. Conversely, data sets from DisProt have larger disordered regions and average region lengths along with a larger fraction of disordered residues. Furthermore, the data set derived from MOBI^4^ and the CheZOD data base have clearly the smallest average region lengths. The CheZOD database is a roughly equal mixture of overall structured proteins and primarily disordered proteins leading to an average disordered region length in between the two extremes.

It is expected that disorder characteristics for the data sets used to train the different prediction methods (see Supp. Table S3) would be reflected in the performance of the resulting disordered predictions. We tested this here by evaluating the performance of the disorder prediction methods on different subsets of the CheZOD database. This analysis was also used to compare the performance of the tested prediction methods, as a good method should be accurate for all possible classes of disordered proteins.

### Prediction accuracy dependence on content of disorder

First, the CheZOD database was split into two parts; one containing those proteins having more than 50% disordered residues, and the other containing those having fewer than 50% disordered residues. The performance on the subsets was assessed by calculating the Pearson correlation between Z-scores and estimated disorder probability, as described in the main text. It was found (see Supplementary Figure S8), that the ranking of the methods was generally preserved. For example, the highest-ranked methods perform better on the mostly ordered dataset whereas the lower ranked methods perform better on the mostly disordered dataset. Furthermore, on average the prediction methods display a lower correlation for the mostly disordered dataset, reflecting that these methods were all trained on data sets with a larger content of ordered residues and are therefore more prone to produce false negatives.

### Region-size dependent prediction accuracy

The ability of the disorder predictors to accurately identify disordered regions of different sizes (lengths) was tested by progressively removing increasingly longer disordered regions from the CheZOD data base and calculating the Pearson Correlation for the remainder (see Supplementary Figure S8). It was generally observed that the correlation rises uniformly upon removing increasingly longer disordered regions. This is consistent with the removal of potential false negative predictions, as discussed above. Data sets from X-ray structures contain much shorter stretches of disorder compared to data from the DisProt database, as already alluded to above. This is mirrored in the finding that the ESpritz^10^ methods experience the largest increase in correlation upon removing the smallest disordered region for the version trained with DisProt and the smallest increase when trained with X-ray (Supp. Fig. S8). Interestingly, several methods give more uniform predictions, whereas other methods fluctuate between high and low probabilities (see e.g. Supplementary Figure S1), which would influence the ability to accurately detect small regions of disorder. This was tested systematically by calculating the predicted average region length, *R’*, as in Eq. (S2) based on fluctuations between high and low probabilities as described above. It is seen that the two binary methods, along with MFDp2^6^ and ESpritz_Disprot^10^, display the largest values for *R’*, while DISOPRED2^7^ and the DisEMBL^8^ methods have the smallest (see Supplementary Figure S9). Indeed, as expected, MFDp2 and ESpritz_Disprot have the largest increase in correlation upon removing the smallest disordered regions from the evaluation, and, conversely, the DisEMBL methods and DISOPRED2 have some of the smallest increases (Supp. Fig. S8). When comparing the values of *R’* to the observed average region lengths for the different data sets (Supp. Table S3) it is clear that the predicted values are generally closer to the observed for CheZOD and MOBI^4^ by NMR (Supp. Fig. S9). The methods with high *R’* mentioned above are closer to the value for DisProt. In particular ESpritz_DisProt, ESpritz_Xray, and ESpritz_NMR^10^ have the decreasingly lower values for *R’*, respectively, reflecting the characteristics of the data set used for training. In contrast, IUPred_long^9^ have only slightly larger R’ compared to IUPred_short. We also note that the application of larger window sizes for the application of sequence features for training would lead to larger values of *R’*.

Another important property for a predictor is the ability to accurately predict the position of the borders between the ordered and disordered regions. This was tested systematically here by evaluating the average border position prediction error, Δ*B* (Eq. (S4)). Statistically, a method with more fluctuations, e.g. smaller average predicted region length, *R’*, as discussed above, would have a lower error, Δ*B*, and therefore we analyzed the combination of these two values. As expected the methods with lower *R’* have consistently smaller Δ*B* and, hence, formally, a better prediction of border positions (see Supplementary Figure S9).

**Supplementary Figure S1**: **Example disorder profiles** **and predictions** for 5 proteins showing Z-scores a black broken line. The estimated disorder probabilities are shown as colored lines for as 15*(1-p) for the 7 best unique methods (SPOT-disorder (red), AUCpreD (grey), MFDp2 (blue), MetaDisorderMD (light blue), PrDOS (magenta), IUPred_long (yellow) and DISOPRED3 (green). The corresponding BMRB id's containing the assigned chemical shifts are provided in the subpanel titles.

**Supplementary Figure S2:** **Receiver Operating Characteristics (ROC) curves** for the true positive rates vs. false positive rates of detecting missing residues in X-ray structures (REMARK 465) for two thresholds of the CheZOD Z-score. Lower values of Z-scores predict missing residues. The blue and the green curve represent the full (222 entries) and the reduced (90 entries) sets of X-ray structures, respectively. The black dashed diagonal line is shown for reference and the area under the ROC curve (AUC) is indicated on the top of the diagram. The blue dots correspond to a cutoff Z-score = 8.0.


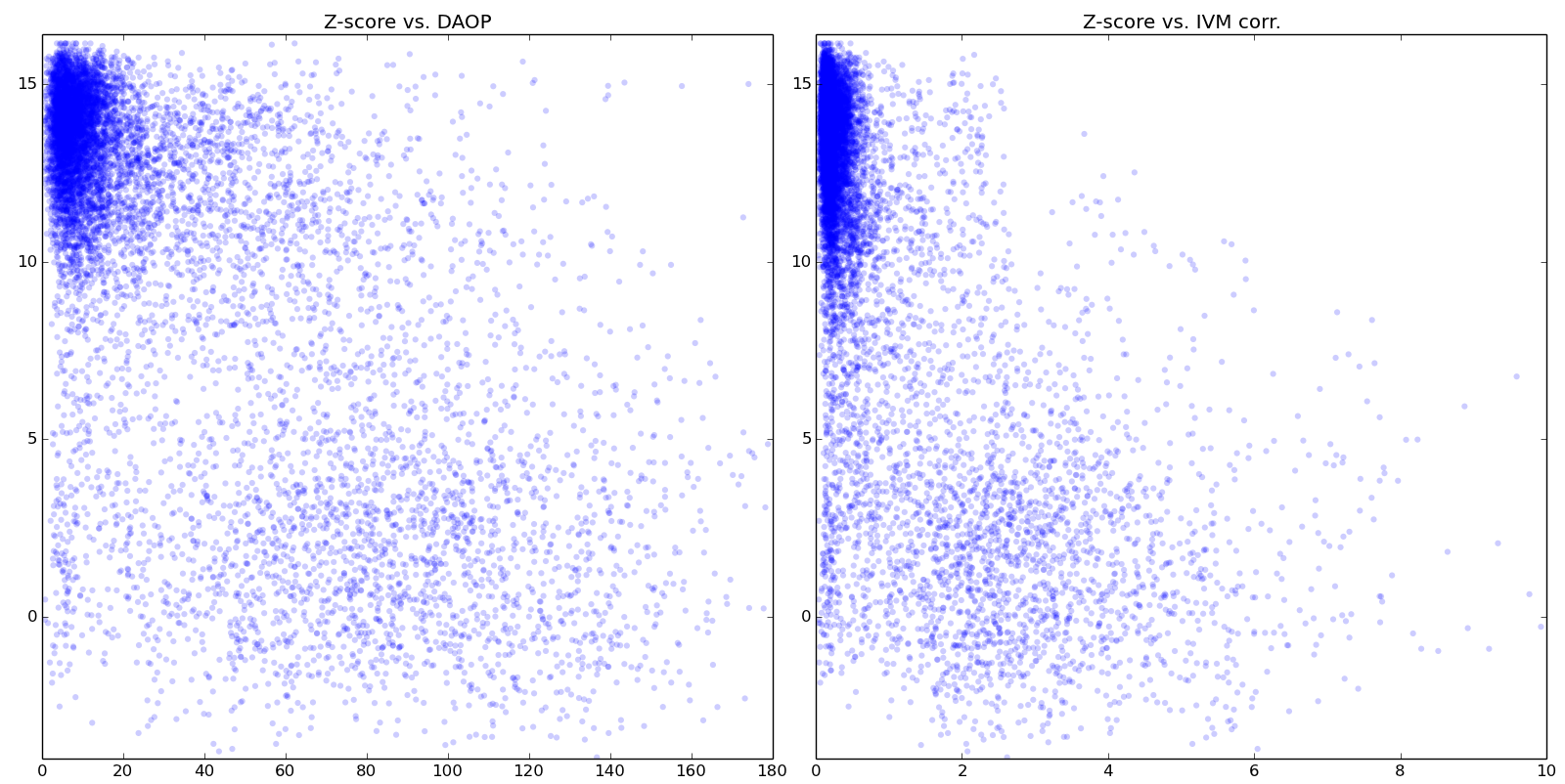


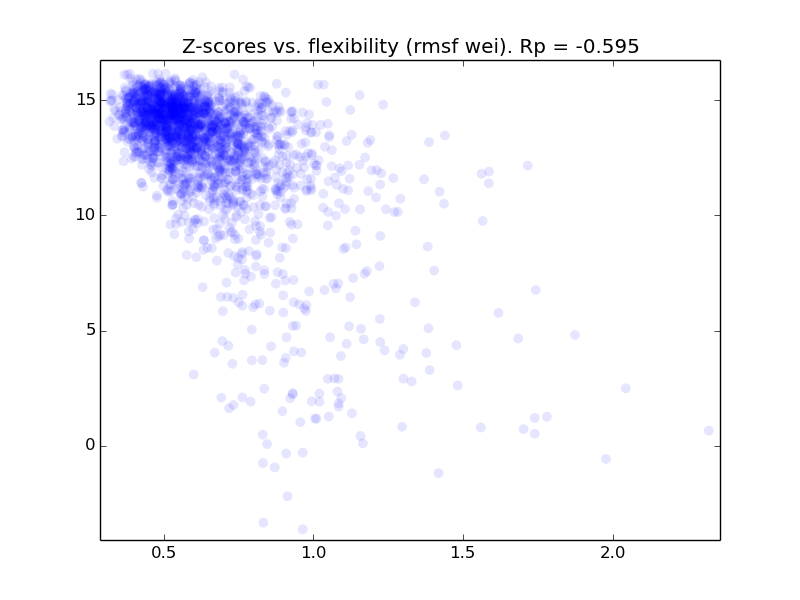


**Supplementary Figure S3:** **Z-scores vs. structural variation.** Residue dihedral angle, coordinate variation, t (top left, Eq. 6 Methods) and s (top right, Eq. 3 Methods), respectively, for the 100 most unstructured entries in the set of protein structures determined by NMR. Bottom: Z-scores vs. rmsf in MD-trajectory in a set of 23 proteins (2301 residues) both in the Dynameomics database and the RefDB database having all backbone chemical shift types assigned.


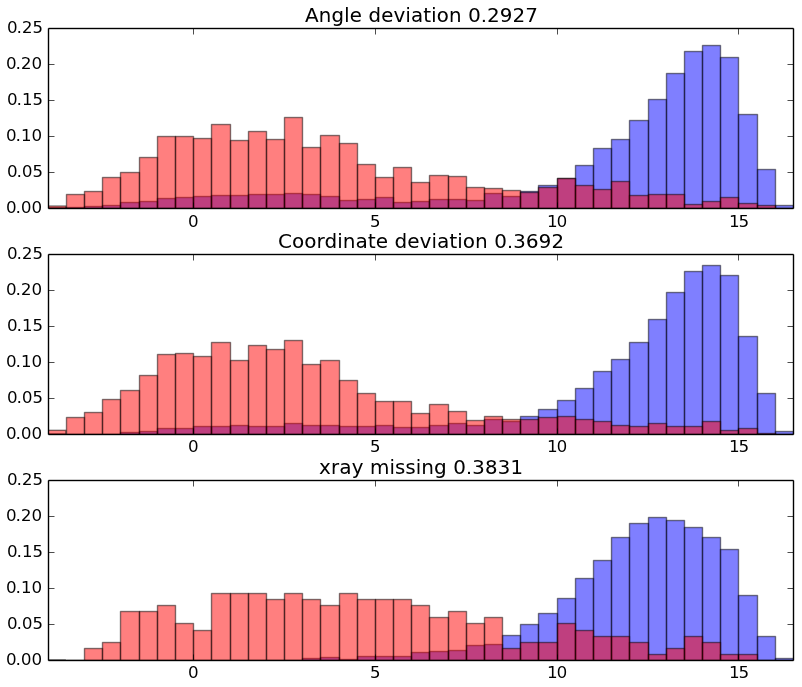


**Supplementary Figure S4**:  **Histogram showing normalized frequencies of Z-scores for split criteria**. top) Angle deviation, t > 75° (red) and t < 75° (blue). middle) Coordinate deviation, s > 1.5Å (red) and s < 1.5 Å (blue). bottom) Missing coordinates in X-ray structures (remark 465) (red), not missing (blue). The Jensen-Shannon divergence, JSD (Eq. (8) Methods), for the similarity of distributions is provided in the title.

**Supplementary Figure S5:** **CheZOD Z-score (green) sequence profiles and local order** **parameters**, 15*S and 15*T (Eq. 7 Methods) for coordinate (red) and dihedral angle (blue) variation, respectively. Data is shown for 9 proteins with mixed secondary structure and flexible loops and/or ends. Proteins with PDB/BMRB ids (from top to bottom panel): 2AFJA/6311, 2BYEA/6624, 2ADZA/6753, 2HDMA/15110, 1WILA/10013, 1TBAA/4223, 1L3GA/4256, 1M3VA/5309, and 1XU6A/6419.

**Supplementary Figure S6:** **Ranking of disorder prediction methods** according to Spearman rank correlation coefficient between estimated disorder probability and Z-score. Colors are the same as in Figure 3 in the main text.

******

**Supplementary Figure S7**: **Ranking of disorder prediction methods** according to AUC for the ROC curves for using estimated disorder probability put predict Z-score under/above the threshold Z = 8. Colors are the same as in Figure 3 in the main text.

**Supplementary Figure S8: Performance on subsets of the CheZOD database**. (left) Pearson correlation of all real-valued methods derived for subsets of the CheZOD database with mostly structured proteins (less than 50% disorder residues, f_IDR_ < 0.5) and mostly disordered proteins (f_IDR_ > 0.5). (right) Pearson correlation for the stripped CheZOD database after removing all data corresponding to residues within short disordered regions with the disorder region (DR) length shown on the x-axis. The data points are connected with lines to enhance visual guidance. The method name is given on the broken line connecting the two panels (the data points for “full set” in the left panel are duplicates of the corresponding data points for DR length = 0 in the right panel). The colors are the same as introduced in Figure 3 main text. The average of all methods are shown for reference with a black broken line. See also Supplementary Discussion.

**Supplementary Figure S9: Prediction of borders between ordered and disordered regions.** Average predicted region length (R’, see Supplementary Discussion and Eq. (S2)) as a function of the average border position prediction error, ΔB, Eq. S4. The observed average region lengths are highlighted with black lines for different experimental data sets as presented in Supplementary Table S3. Each predictor is shown with a circle using the same colors as in Fig. 3 main text. The vertical scale is logarithmic.

**Supplementary Figure S10: Optimization of threshold for binary classification**. Mathews Correlation Coefficient (MCC) as a function threshold for Z-score for all methods. Colors as introduced in Figure 3 in the main text.

**Supplementary Table S1: cross-comparison of predictor data agreement**

| Data source^a^ | Method | *AUC* (present) | *MCC*  (present) | *AUC* (source) | *MCC*  (source) | *AUC* (diff) | *MCC*  (diff) |
| --- | --- | --- | --- | --- | --- | --- | --- |
| DisProt | MFDp2 | 0.853 | 0.578 | 0.677 | 0.343 | 0.176 | 0.235 |
| DisProt | AUCpreD | 0.865 | 0.540 | 0.728 | 0.400 | 0.137 | 0.140 |
| DisProt | MetaDisorder | 0.865 | 0.541 | 0.570 | 0.388 | 0.295 | 0.153 |
| DisProt | MetaDisorderMD | 0.853 | 0.519 | 0.595 | 0.382 | 0.258 | 0.137 |
| DisProt | MetaDisorderMD2 | 0.852 | 0.540 | 0.693 | 0.359 | 0.159 | 0.181 |
| DisProt | MetaDisorder3D | 0.727 | 0.333^e^ | 0.611 | 0.032 | 0.116 | 0.301 |
| DisProt | IUPred_long | 0.834 | 0.512 | 0.757 | 0.377 | 0.077 | 0.135 |
| DisProt | IUPred_short | 0.822 | 0.429 | 0.755 | 0.360 | 0.067 | 0.069 |
| DisProt | ESpritz_Xray | 0.791 | 0.286 | 0.758 | 0.341 | 0.033 | -0.055 |
| DisProt | ESpritz_DisProt | 0.748 | 0.412 | 0.800 | 0.410 | -0.052 | 0.002 |
| DisProt | DISPROT (VSL2b) | 0.808 | 0.466 | 0.767 | 0.339 | 0.041 | 0.127 |
| DisProt | s2D | 0.797 | 0.421 | 0.721 | 0.248 | 0.076 | 0.173 |
| DisProt | DisEMBL_hotloops | 0.702 | 0.239^f^ | 0.659 | 0.196 | 0.043 | 0.043 |
| DisProt | DisEMBL_remark465 | 0.737 | 0.284 | 0.704 | 0.268 | 0.033 | 0.016 |
| DisProt | DISOPRED3 | 0.833 | 0.451 | 0.766 | 0.369 | 0.067 | 0.082 |
| DisProt | **Average** | 0.805 | 0.436 | 0.700 | 0.317 | **0.103** | **0.116** |
| X-ray | MetaDisorderMD^b^ | 0.853 | 0.519 | 0.840 | 0.340 | 0.013 | 0.179 |
| X-ray | PrDOS^b^ | 0.836 | 0.409 | 0.910 | 0.530 | -0.074 | -0.121 |
| X-ray | DISPROT (VSL2b)^d^ | 0.808 | 0.466 | 0.810 | 0.260 | -0.002 | 0.206 |
| X-ray | RONN^d^ | 0.804 | 0.455 | 0.760 | 0.220 | 0.044 | 0.235 |
| X-ray | DisEMBL_remark465^d^ | 0.737 | 0.284 | 0.790 | 0.310 | -0.053 | -0.026 |
| X-ray | ESpritz_Xray^b^ | 0.791 | 0.286 | 0.860 | 0.360 | -0.069 | -0.074 |
| X-ray | ESpritz_NMR^b,c^ | 0.797 | 0.374 | 0.643 | 0.226 | 0.154 | 0.148 |
| X-ray | ESpritz_DisProt^b,c^ | 0.748 | 0.412 | 0.530 | 0.069 | 0.218 | 0.343 |
| X-ray | IUPred_long^b,c^ | 0.834 | 0.512 | 0.573 | 0.159 | 0.261 | 0.353 |
| X-ray | IUPred_short^d^ | 0.822 | 0.429 | 0.780 | 0.310 | 0.042 | 0.119 |
| X-ray | DISOPRED3^b^ | 0.833 | 0.451 | 0.897 | 0.531 | -0.063 | -0.080 |
| X-ray | **Average** | 0.803 | 0.415 | 0.750 | 0.278 | **0.042** | **0.116** |

^a^ Data from Disprot^10^ and missing densities in X-ray structures (see footnotes below)

^b^ From CASP10 evaluation^11^

^c^ Data from D^2^P^2^ from a 51 proteins subset of CASP10

^d^ Data from^12^

^e^ The threshold, *p* = 0.30, was used for assigning binary predicted order/disorder as estimated from the apparent split in the scattering of the data in Figure 2 in the main text

^f^ The threshold, *p* = 0.12, was used for assigning binary predicted order/disorder as is default on the DisEMBL server (<http://dis.embl.de/>)

Supplementary Table S2: Detailed statistics of binary classification metrics for all disorder prediction methods^a^

| Method | TPR | TNR | FPR | FNR | prec | Acc |
| --- | --- | --- | --- | --- | --- | --- |
| MFDp2 | 0.8528 | 0.7275 | 0.2725 | 0.1472 | 0.8599 | 0.7901 |
| MetaDisorderMD2 | 0.7688 | 0.7955 | 0.2045 | 0.2312 | 0.8806 | 0.7821 |
| MetaDisorderMD | 0.7246 | 0.8227 | 0.1773 | 0.2754 | 0.8891 | 0.7736 |
| MetaDisorder | 0.8221 | 0.7287 | 0.2713 | 0.1779 | 0.8560 | 0.7754 |
| MetaDisorder3D | 0.5841 | 0.7674 | 0.2326 | 0.4159 | 0.8312 | 0.6757 |
| SPOT-dis | 0.7067 | 0.8757 | 0.1243 | 0.2933 | 0.9177 | 0.7912 |
| AUCpreD | 0.7154 | 0.8555 | 0.1445 | 0.2846 | 0.9066 | 0.7854 |
| PrDOS | 0.5333 | 0.8890 | 0.1110 | 0.4667 | 0.9041 | 0.7111 |
| RONN | 0.7387 | 0.7368 | 0.2632 | 0.2613 | 0.8463 | 0.7377 |
| DISpro | 0.3185 | 0.9565 | 0.0435 | 0.6815 | 0.9349 | 0.6375 |
| DISOPRED2 | 0.6420 | 0.6994 | 0.3006 | 0.3580 | 0.8073 | 0.6707 |
| DISOPRED3 | 0.5301 | 0.9207 | 0.0793 | 0.4699 | 0.9292 | 0.7254 |
| ESpritz_NMR | 0.4662 | 0.9087 | 0.0913 | 0.5338 | 0.9092 | 0.6874 |
| ESpritz_Xray | 0.2820 | 0.9597 | 0.0403 | 0.7180 | 0.9321 | 0.6208 |
| ESpritz_DisProt | 0.8208 | 0.5846 | 0.4154 | 0.1792 | 0.7949 | 0.7027 |
| AUCpreD_noEvo | 0.6030 | 0.8664 | 0.1336 | 0.3970 | 0.8985 | 0.7347 |
| DISPROT (VSL2b) | 0.8525 | 0.5986 | 0.4014 | 0.1475 | 0.8064 | 0.7256 |
| IUPred_long | 0.7477 | 0.7887 | 0.2113 | 0.2523 | 0.8741 | 0.7682 |
| IUPred_short | 0.6284 | 0.8249 | 0.1751 | 0.3716 | 0.8756 | 0.7267 |
| Pdisorder | 0.7592 | 0.7128 | 0.2872 | 0.2408 | 0.8383 | 0.7360 |
| DisEMBL_coils | 0.7252 | 0.6016 | 0.3984 | 0.2748 | 0.7812 | 0.6634 |
| DisEMBL_remark465 | 0.4219 | 0.8598 | 0.1402 | 0.5781 | 0.8551 | 0.6409 |
| DisEMBL_hotloops | 0.4320 | 0.8093 | 0.1907 | 0.5680 | 0.8163 | 0.6206 |
| GlobPlot | 0.0200 | 0.9887 | 0.0113 | 0.9800 | 0.7758 | 0.5043 |

^a^ From left right the columns the columns contain values for True Positive Rate, True Negative Rate, False Positive Rate, False Negative Rate, precision and balanced accuracy. The latter two are discussed on previous studies of assessing the performance of predictors, see. e.g. CASP evaluation reports^11^.

**Supplementary Table S3: Disorder characteristics of data sets**

| Data set | *f*_IDR_^a^ | *R*_dis_ (average)^b^ | *R* (Eq. (S2)) | *N* (average)^c^ |
| --- | --- | --- | --- | --- |
| CASP9 | 9.216 | 12.14 | 89.57 | 225.1 |
| CASP10 | 5.920 | 17.21 | 133.53 | 269.9 |
| DM4229 (no X-ray)^d^ | 8.574 | 10.56 | 92.16 | 241.4 |
| DisProtv7Primary^e^ | 17.721 | 47.05 | 170.62 | 427.3 |
| DisProtv7Complement^f^ | 25.739 | 80.27 | 230.15 | 429.4 |
| MOBI (by NMR)^g^ | 16.906 | 8.57 | 36.81 | 79.2 |
| CheZOD (present study) | 66.233 | 24.41 | 23.02 | 111.8 |

^a^ Fraction of disordered residues

^b^ Average length of disordered regions

^c^ Average number of residues

^d^ X-ray data set excluding DisProt entries used to train SPOT-disorder^13^

^e^ DisProt data base version 7.0^5^ (from http://www.disprot.org/assessment) only for the “primary” methods: NMR and X-ray (from )

^f^ DisProt data base version 7.0^5^ (from http://www.disprot.org/assessment) excluding older version entries (used for evaluation of predictors by Necci et al^10^).

^g^ The data set used for training ESpritz_NMR^4,14^.

## References

1 Nielsen, J. T. & Mulder, F. A. A. POTENCI: prediction of temperature, neighbor and pH-corrected chemical shifts for intrinsically disordered proteins. *J Biomol NMR* **70**, 141-165, (2018).

2 Benson, N. C. & Daggett, V. Dynameomics: Large-scale assessment of native protein flexibility. *Protein Sci* **17**, 2038-2050, (2008).

3 van der Kamp, M. W. *et al.* Dynameomics: A Comprehensive Database of Protein Dynamics. *Structure* **18**, 423-435, (2010).

4 Martin, A. J., Walsh, I. & Tosatto, S. C. MOBI: a web server to define and visualize structural mobility in NMR protein ensembles. *Bioinformatics* **26**, 2916-2917, (2010).

5 Piovesan, D. *et al.* DisProt 7.0: a major update of the database of disordered proteins. *Nucleic Acids Res* **45**, D219-D227, (2017).

6 Mizianty, M. J., Peng, Z. & Kurgan, L. MFDp2. *Intrinsically Disordered Proteins* **1**, e24428, (2013).

7 Ward, J. J., Sodhi, J. S., McGuffin, L. J., Buxton, B. F. & Jones, D. T. Prediction and functional analysis of native disorder in proteins from the three kingdoms of life. *J Mol Biol* **337**, 635-645, (2004).

8 Linding, R. *et al.* Protein disorder prediction: implications for structural proteomics. *Structure* **11**, 1453-1459, (2003).

9 Dosztanyi, Z., Csizmok, V., Tompa, P. & Simon, I. IUPred: web server for the prediction of intrinsically unstructured regions of proteins based on estimated energy content. *Bioinformatics* **21**, 3433-3434, (2005).

10 Necci, M., Piovesan, D., Dosztanyi, Z., Tompa, P. & Tosatto, S. C. E. A comprehensive assessment of long intrinsic protein disorder from the DisProt database. *Bioinformatics* **34**, 445-452, (2018).

11 Monastyrskyy, B., Kryshtafovych, A., Moult, J., Tramontano, A. & Fidelis, K. Assessment of protein disorder region predictions in CASP10. *Proteins* **82**, 127-137, (2014).

12 Walsh, I. *et al.* Comprehensive large-scale assessment of intrinsic protein disorder. *Bioinformatics* **31**, 201-208, (2015).

13 Hanson, J., Yang, Y., Paliwal, K. & Zhou, Y. Improving protein disorder prediction by deep bidirectional long short-term memory recurrent neural networks. *Bioinformatics* **33**, 685-692, (2017).

14 Walsh, I., Martin, A. J., Di Domenico, T. & Tosatto, S. C. ESpritz: accurate and fast prediction of protein disorder. *Bioinformatics* **28**, 503-509, (2012).
